# Supplementary material for: Interventions to Promote the Utilization of Physical Health Care for People with Severe Mental Illness: A Scoping Review
Source: Int J Environ Res Public Health. 2022 Dec 22;20(1):126. doi: 10.3390/ijerph20010126 (PMC9819522; doi:10.3390/ijerph20010126)
Supplement: Supplementary file 1 [file ijerph-20-00126-s001.zip › suppl-table-S7_complete-study-characteristics.v2.pdf]

Table S7: Studies investigating interventions to promote utilization of physical health care for people with SMI: Complete study characteristics

Study identi-fication number	Reference	Country	Study Design	• Aim<br>• Setting of Intervention<br>• Sample characteristics[1]	Interventions	Theoretical rationale or model	• Outcome measures<br>• Main results
1	Aftab et al., 2018 [52]	USA	RCT	Aim: To examine the effects of a group-based self-management training on psychiatric symptomatology and diabetic control<br>Context/Setting: Safety-net health system primary care setting<br>Sample: 100 individuals with SMI (schizophrenia, schizoaffective disorder, bipolar disorder or major depressive disorder) and Type 2 diabetes	- 12 weekly group sessions delivered by a nurse educator and a peer educator with SMI and Diabetes<br>- In the following 48 weeks, participants have 10-15min phone maintenance sessions. For the first 3 months every other week, and monthly thereafter<br>- Topics: psychoeducation, better use of health care, problem solving, lifestyle changes and self-management	Adaptation of evaluated programs (Life Goals Program and Diabetes Awareness and Rehabilitation Training); Applying principles of social cognitive theory	Outcome measures:<br>- Mental illness symptom severity<br>- Functioning<br>- General medical health<br>- Diabetes control (HbA1c - Levels)<br>- BMI<br>- Blood pressure<br>- Diabetes knowledge<br>- Diabetes self-care<br>Main findings:<br>- Improvement in depression, global psychopathology and functioning<br>- Less deterioration in diabetes control only in subgroup of participants with reasonably good baseline diabetes control<br>- Improvement in diabetes knowledge<br>- No improvement in general medical health, BMI, blood pressure and diabetes self-care

2	Alexopoulos et al., 2014; Lawless et al., 2016; Sajatovic et al., 2017; Sajatovic et al., 2011 [53,86,95,96]	USA	RCT	<u>Aim:</u> To investigate the effects of a personalized intervention for depression and COPD (PID-C) vs. usual care (UC) on individuals with COPD and major depression<br><u>Context/Setting:</u> Recruitment in rehabilitation clinic<br><u>Sample:</u> 67 COPD-Patients with unipolar major depression	- Intervention started at the end of hospitalization<br>- 9 x 30 min sessions with care managers over the course of 26 weeks<br>- 1st session before discharge, 2nd to 9th session at the patients' home<br>- Mobilizing patients for treatment engagement of both diagnoses<br>- Contact of patients' physicians for treatment recommendations<br>- Targeting treatment obstacles to make use of health care<br>- Support in COPD exercises and AD adherence	Evaluated intervention, based on 'Theory of reasoned action"	<u>Outcome measures</u>:<br>- Depressive and anxiety symptoms,<br>- Dyspnea-related disability,<br>- Medical burden (interviewer-assessment),<br>- Adherence to exercise (interviewer-assessment),<br>- Executive dysfunction (assessment)<br>- Neuroticism<br>- Social network and support<br>- Adequacy of antidepressant prescription (interviewer-assessment)<br><u>Main findings:</u><br>- Improvement of both conditions in PID-C group greater than in UC<br>- Antidepressant adherence and COPD exercising led to an interaction spiral of improvement for outcomes<br>- Positive interaction mediated through adherence of adequate AD adherence and exercise adherence
3	Aragonès et al., 2012 [54]	Spain	Cluster RCT	<u>Aim:</u> To examine the effectiveness of a multi-component programme for	- Intervention duration of 12 months<br>- Monthly structured doctor and nurse	Adaption of the chronic care model to primary care in	<u>Outcome measures:</u><br>- Severity of depression symptoms

				managing depression in primary care<br>Context/Setting: 20 primary care centers<br>Sample: 189 individuals diagnosed with a major depressive episode	visits until remission of depressive episode; Visits every two or three months in maintenance stage during study period<br>- Clinical training and support tools for decisions taken by primary-care doctors and nurses<br>- Case-management by primary care nurses<br>- Impovements in the primary care-psychiatry information interface<br>- One-to-one psychological and educational support to overcome stigma and to enhance patient empowerment	the Spanish public health system	- treatment response and remission rates<br>- Health related quality of life<br>- Continuity of antidepressant treatment (administrative data)<br>- Number of primary care and psychiatric visits for depression or related health problems (administrative data)<br>Main findings:<br>- Higher depressive symptom and global psychopathology decrease<br>- Health related quality of life: improvement in mental health but not physical health component<br>- Increased visits with nurses<br>- Higher adherence the antidepressant medication
4	Bartels et al., 2013 [55]	USA	Pre-post pilot study	Aim: To evaluate the feasibility and potential effectiveness of a collaborative activation training for individuals with SMI and their primary care providers	- 9 group-based 90-min sessions delivered weekly over 2 months<br>- Group-based educative Skills Training for patients: a) basics of heart	Components of an evidence-based health care management intervention	Outcome measures:<br>- Patient activation<br>- Perceived efficacy in patient-physician interaction<br>- Health care decision making and information seeking

				Context/Setting: Community-based mental health center; Primary care practices<br>Sample: 17 Individuals with SMI (schizophrenia spectrum disorder, bipolar disorder, or major depression) age >= 50 with cardiovascular risk factors; 6 primary care providers (internists, family physicians, nurse practitioners)	health, b) personal health assessment, c) setting achievable lifestyle goals, d) making the most of a health care visit, e) communicating effectively with health care providers, f) getting help with medical visits from family members<br>- Co-occurence of peer-specialists and health-care professionals<br>- Provider training to improve knowledge, communication skills and collaboration in SMI and cardiovascular risk		- Social skills performance (assessment through role play),<br>- Participant experiences and satisfaction<br>Main findings:<br>- Significant improvement in patient activation<br>- ignificant improvement in communication skills<br>- Self-management, self-efficacy and patient activation focus seems to bridge gap between physical health care and people with SMI
5	Bartels et al., 2014 [56]	USA	RCT	Aim: To evaluate one, two and three-year outcomes of a combined psychosocial skills training and preventive health care intervention for older persons with SMI<br>Context/Setting: Mental health centers and senior housing settings in the	- Weekly group skills training over one year and one-year maintenance phase with monthly booster sessions. Contains social skills training including the topics: Communicating effectively;	Based on conceptual model of psychosocial rehabilitation and health management for older people with SMI	Outcome measures:<br>- Community living skills (self-report and assessment based)<br>- Psychiatric symptoms<br>- Health status (self-report and interview based assessment)<br>Main findings:<br>- Improved community living skills (greater self-efficacy

				community; Individuals were living on their own.<br>Sample: 90 adults (age >= 50) with SMI (schizophrenia, schizoaffective disorder, bipolar disorder or major depression) and persistent physical health impairments.	Friendships; Leisure Time; Healthy living; Medication use; Health care visits optimization. Practical skills training through community trips twice a month..<br>- Monthly meetings with a nurse who helps facilitate preventive screening, advance care planning, and coordination of primary care visits.<br>- Group skills training leaders and health nurses coordinated components together<br>"		and decreased overall severity of psychiatric symptoms) at 3-year follow-up<br>- Greater receipt of preventive health screening<br>- Higher number of identified medical diseases.<br>- Fewer hospitalizations and ER visits<br>- Improved quality of preventive health care without improvement of physical health status.
6	Bartels et al., 2014 [57]	USA	Pilot-RCT	Aim: To evaluate the feasibility and effectiveness of a self-management training for psychiatric and general medical illness including embedded nurse care management for individuals with SMI	- ten-module curriculum, delivered for eight-months period during weekly sessions<br>- Contains self-management based psychoeducation to psychiatric conditions	Adaption and extension of an evidence-based program for psychiatric illness self-management (IMR: Illness Management and Recovery) to chronic	Outcome measures:<br>- Participant attendance (participation data)<br>- Self-management regarding illnesses (self-report & interview)<br>- Communication in medical encounters (self-reports by interviewers)

				<u>Context/Setting:</u> Two community mental health centers<br><u>Sample:</u> 36 adults (age >= 50) with SMI (schizophrenia, major depression or bipolar disorder) and persistent physical health condition.	(CBT-approaches to increase medication adherence; Training in relapse prevention, Instruction about coping skills and social skills training) and general medical condition (Application of same skills to medical condition, individually tailored).<br>- Specialist with Masters in Social Work; Nurse health care manager facilitates preventive and ongoing care through meetings twice per month.<br>- Intervention also delivered in group setting	general medical conditions	- Use of acute care services (self-reports by interviewers)<br><u>Main findings:</u><br>- Improvements in self-management psychiatric illness and diabetes but not for general medical conditions<br>- Greater participation in primary health care encounters.<br>- Fewer psychiatric or general medical hospitalizations
7	Battersby et al., 2018 [58]	Australia	Study Protocol for phase III randomized effectiveness trial	<u>Aim:</u> To describe the construction and evaluation of a program which reduces CVD risk and improves quality of life for individuals with SMI	- Intervention duration: 12 months; 24 months follow-up<br>- Intervention frequency: weekly to every three months?	Person centered process underpinned by cognitive and behavioral theory and motivational techniques;	<u>Intended outcome measures:</u><br>- Absolute cvd risk (self-report by interviews/clinical measures)<br>- Health related quality of life<br>- Quality adjusted life years

				Context/Setting: Public psychiatric service. Ongoing support via face-to-face or remotely.<br>Sample: Persons with SMI (schizophrenia, schizoaffective disorder, bipolar disorder or depressive psychosis) with age >= 30 and at least one CVD risk factor.	- 6 follow-up meetings with trial nurse, which helps patients with their care-plan: Monitoring of outcomes of care-plan; Motivation and problem-solving approaches to assist patients in goal-achievement; Care-coordination; Support to access existing structures; Initiation adherence to other physical health treatments<br>- Risk assessment: Two assessments for problem identification; Clinical assessment of triglycerides and diabetes and goal setting.<br>- Intervention delivered individually and in group setting for weight reduction	consistent with chronic care model principles of self-management support and recovery oriented care.	

8	Bjorkman und Hansson, 2000 [59]	Sweden	One group pre-post design.	Aim: To evaluate the effects of case management services on health care service use of individuals with long-term SMI<br>Context/Setting: 10 different case-management services. Case management took place 70% of the time outside the case management office in clients home.<br>Sample: 176 clients ("long-term mentally ill"; more then half of participants with diagnosis of psychosis) of different case-management services	- Intervention duration: 18 months<br>- Intervention frequency not specified<br>- Social workers/psychiatric nurses working as case managers: Mobilization of social network; Crisis intervention; Referrals and links to present community resources; Support in daily living	Heterogenous case-management models.<br>No precise intervention because of 10 different settings	Outcome measures:<br>- Use of health care services (psychiatric inpatient care, psychiatric day care, psychiatric outpatient care, primary care, somatic inpatient care, somatic outpatient care other than primary care) (administrative data)<br>Main findings:<br>- Reduction in inpatient psychiatric care use<br>- No differences in use of primary health care (during 18-month period prior to and after clients admission into case management service)
9	Blank et al., 2014 [60]	USA	RCT	Aim: To test the effectiveness of a community-based advanced practice nurse intervention to promote adherence to HIV and psychiatric treatment regimens.<br>Context/Setting:Meetings with individuals were at their home or another location of their choosing. Recruitment at somatic treatment site	- At minimum one visit per week for 12 months. Advanced practice nurses held adaptive treatment: In-home consultations and coordination of medical and mental health services.<br>- Intervention contained psychoeducation and	Based on the program of Assertive Community Treatment (ACT); Services are delivered in the community instead of clinics as well as use of advanced practice nurses	Outcome measures:<br>- Health related quality of life<br>- HIV testing and viral load<br>Main findings:<br>- Intervention improved biomarkers for viral load and health related quality of life indicators.<br>- Adherence behavior in intervention group continued after withdrawal of intervention

				<u>Sample:</u> 128 individuals with SMI (not specified) and HIV	coordination of physician appointments.<br>- Escalation strategy: Tracking of medication adherence and adjustment of intervention		
10	Broughan et al., 2021; McCombe et al., 2019 [61,88]	Ireland	Qualitative study	<u>Aim:</u> To evaluate a keyworker-mediated intervention promoting physical health among first episode psychosis patients<br><u>Context/Setting:</u> Three mental health services<br><u>Sample:</u> 18 individuals with first-episode psychosis	- Intervention duration: 3 months<br>- Intervention frequency not specified<br>- Regular bidirectional meetings between physical health keyworkers and participants (6 weeks, disrupted due to covid-19 pandemic)<br>- Identification of health issues and concerns<br>- Conduct of physical health and lifestyle assessments<br>- Evaluation of participants' health goals	*Implementation framework:* Framework for design and evaluation of complex interventions to improve health; Theoretical framework for healthcare interventions<br>*Intervention:* Combining empirical evidence of keyworker-mediated interventions and physical health interventions for people with first-episode psychosis	<u>Outcome measures:</u><br>- Feasibility (participation data),<br>- Acceptability (participation data and semi-structured interviews)<br>- View of participants on role of the physical health keyworker<br><u>Main findings:</u><br>- Physical health keyworker support and encouragement was perceived as helpful by participants<br>- Care coordination to GP could be intensified<br>- Pandemic restrictions interrupted some of the interventions' core components but telemedicine interventions showed good acceptability

11	Cabassa et al., 2018 [62]	USA	Mixed-methods single group design.	Aim: To examine the feasibility, acceptability, and initial impact of an health intervention for Hispanics with SMI.<br>Context/Setting: Public outpatient mental health clinic.<br>Sample: 34 Hispanic individuals with SMI (schizophrenia, schizoaffective disorder, major depressive disorder, bipolar disorder, major depressive disorder with psychotic features) and at least one CVD risk factor (BMI >= 25, diabetes mellitus, smoking, hypertension, or hyperlipidemia).	- Monthly individual 60-minutes sessions delivered by a social worker for 12 months.<br>- Focus on care coordination between mental health and primary care providers and patient activation for physical health issues (developing knowledge and skills).<br>- Health care managers serve as bridge to reduce fragmentation of care by sharing medical information across providers, monitoring patients' health, and alerting providers when preventive primary care is needed.	Cultural adaptation of PCARE, an evidence-based health care manager intervention delivered by registered nurses.	Outcome measures:<br>- Feasibility and acceptability: recruitment rate, session attendance, assessment completion rates, satisfaction<br>- Patient activation (self-report with interview)<br>- Self-efficacy (self-report with interview)<br>- Participants' perspectives on quality and patient-centeredness of chronic illness care<br>- Health-related quality of life<br>- Receipt of preventive primary care services (based on administrative data)<br>- Mental health diagnoses (based on administrative data)<br>- Physical health conditions (based on administrative data)<br>- Smoking status (based on administrative data<br>Main findings:<br>- Significant improvements of patient activation, self-efficacy, and quality of illness care

							- Significant improvement of receipt of preventive primary care services<br>- No significant improvements of chronic illness care received from primary care physicians<br>- No improvement in health-related quality of life
12	Chwastiak et al., 2018 [63]	USA	Randomized controlled pilot study	Aim: To pilot test a community mental health center based collaborative care model to treat type II diabetes among outpatients with SMI<br>Context/Setting: Community mental health centers<br>Sample: 18 Individuals with SMI diagnosis (schizophrenia, schizoaffective disorder, bipolar disorder, or major depressive disorder) and diagnosis of type II diabetes.	- Intervention was provided by a team of a nurse care manager, a psychiatrist, an advanced practice registered nurse and an endocrinologist consultant.<br>- Initial 60-minutes nurse care manager visit, twelve weeks weekly 30-minutes chronic illness self-management support (medication adherence, healthy nutrition, and regular physical activity), then monthly visits for 6 months.<br>- Use of motivational interviewing and	Intervention based on principles of the chronic care model and an evidence-based intervention (TEAMcare)	Outcome measures:<br>- HbA1c-levels<br>- BMI<br>- Smoking status<br>- Psychiatric symptoms<br>Main findings:<br>- Clinically and statistically significant change in Hb1A1c-levels at end of the three months intervention.<br>- Decrease in BMI

					behavioral activation techniques for care coordination with primary care and specialty medical providers.		
13	Cook et al., 2020 [64]	USA	RCT	<u>Aim:</u> To assess the longitudinal effectiveness of a peer-delivered medical illness self-management program for adults with SMI<br><u>Context/Setting:</u> Three community mental health agencies<br><u>Sample:</u> 73 adults with SMI (schizophrenia, bipolar disorder, major depressive disorder) and the presence of a medical condition or health impairment the participant wanted to adress	- Participants met once a week in a peer specialists-led group; First three sessions lasted 2.5 hours (How to develop wellness goals and how to formulate weekly action plans); 8 group sessions of 90 minutes duration (Health and recovery topics; Power of peer support; Effective communication with health providers; Taking care of oneself; Managing chronic conditions); 12th session was for graduation.)<br>- Also individual weekly meetings with a peer specialist; First three sessions of 45	Combining evidence of self-management programs and peer-support in the intervention	<u>Outcome measures:</u><br>- General medical self-management<br>- WHO Question on general health<br>- Hopefulness<br>- Paid employment of past 90 days<br>- Participant satisfaction<br><u>Main findings:</u><br>- Significantly greater improvement in the primary outcome of general medical self-management and self-rated general medical health<br>- Improvement in self-reported hope<br>- Twice as many participants worked for pay in intervention condition at the end of study (38% were referred to employement services)

					min, then 9 sessions of 15 min including goal setting and action planning as well as care coordination support<br>- 3 months of intervention duration<br>- 3 months of follow-up measurement		
14	Daumit et al., 2020 [65]	USA	RCT	<u>Aim:</u> To determine the effectiveness of an 18-month multifaceted intervention incorporating behavioral counseling, care coordination, and care management for overall cardiovascular risk reduction in adults with SMI<br><u>Context/Setting:</u> Outpatient community mental health programs.<br><u>Sample:</u> 132 individuals with SMI (schizophrenia, schizoaffective disorder, bipolar disorder, major depression) and at least one cardiovascular risk factor	- Intervention duration: 18 months<br>- Use of solution-focused therapy and motivational interviewing<br>- Intervention delivered by health coaches and a health nurse<br>- Cardiovascular risk reduction education (health nurse); Health coaching sessions (health coach); Collaboration with physicians; Coordination with mental health staff	Intervention design based on self-management concepts and social cognitive theory; Integration of care coordination and care management approaches.	<u>Outcome measures:</u><br>- 10-year probability of cardiovascular disease event (self-report and physiological data)<br>- Blood pressure<br>- Bloodwork<br>- BMI<br>- Smoking status<br><u>Main findings:</u><br>- Significant reduction of the estimated 10-year risk of a cardiovascular event<br>- Significantly reduced rates of tobacco smoking<br>- No significant between-group differences in other CVR measures

					- Health coaching sessions were 20 to 30 minutes long, weekly for 6 months, every 2 weeks thereafter<br>- Setting of health goals and how to achieve them		
15	Druss et al., 2018; Druss et al., 2010 [66,68]	USA	RCT	<u>Aim:</u> To examine the effectiveness of a peer-led program for self-management of general medical conditions for individuals with SMI<br><u>Context/Setting:</u> One urban and two suburban community mental health centers<br><u>Sample:</u> 198 individuals with SMI (schizophrenia, schizoaffective disorder, bipolar disorder, major depression, obsessive-compulsive disorder, or posttraumatic stress disorder, with or without a comorbid substance use disorder) and one or more chronic general medical illnesses	- Intervention duration: 6 months<br>- Intervention frequency: weekly to monthly<br>- Six group sessions (2.5h) led by two peer-specialists.<br>- Sessions provide information about chronic general medical conditions, use of action planning, healthy diet and exercise on a budget, communicating with health-care providers, medication and other treatments, and	Health and recovery peer program (HARP), grounded on the Chronic Disease Self-Management Program (CDSMP) and adapted to the specific needs of patients with SMI	<u>Outcome measures:</u><br>- Health related quality of life<br>- Patient activation<br>- Diet<br>- Medication adherence<br>- Access to primary care<br>- Mental health recovery<br><u>Main findings:</u><br>- Improved physical and mental health-related quality of life<br>- Short-term improvements in patient-activation, no longer significant after 6 months

					working across the health system.<br>- One-on-one peer coaching were held between group sessions to reinforce learnings		
16	Druss et al., 2010 [67]	USA	RCT	Aim: To test a population-based medical care management intervention to improve primary medical care for individuals with SMI<br>Context/Setting One urban community mental health center<br>Sample: 205 individuals with SMI (schizophrenia, schizoaffective disorder, bipolar disorder, depression, PTSD)	- Intervention duration: 12 months<br>- Intervention frequency: not specified<br>- Registered nurses helped patients overcome their own-, provider-, and system-level barriers to primary medical care.<br>- Provision of information about patient's medical conditions, available medical providers and upcoming appointments.<br>- Use of motivational interviewing techniques supported patient self-management skills;	Based on evidence of care management and care coordination approaches	Outcome measures:<br>- Quality of primary/preventive care (review of health charts)<br>- Quality of cardiometabolic care (review of health charts)<br>- Presence of a primary care provider (self-report)<br>- Health-related quality of life<br>Main findings:<br>- More than doubled rate of receipt of evidence-based preventive medical services in intervention group<br>- Significant improvement on mental health but not physical health related quality of life

					Use of action plans; Coaching to interact more efficiently with providers; Accompanied patients as needed to visits		
17	Goldberg et al., 2013 [69]	USA	RCT	<u>Aim:</u> To examine the effectiveness of a chronic disease self-management program for individuals with SMI<br><u>Context/Setting:</u> Four mental health settings (one outpatient clinic and three psychiatric rehabilitation day programs)<br><u>Sample:</u> 32 individuals with SMI (schizophrenia or bipolar disorder with psychotic features) and at least one chronic general medical condition.	- Intervention duration: 5 months<br>- Delivery of intervention by a mental health peer or a mental health provider and a peer co-leader.<br>- 13 weekly 60 - 75 minute sessions; Between sessions, individuals were telephoned by peer facilitators to review their progress<br>- Two booster sessions after the end of intervention<br>- Intervention included basic self-management strategies, specific disease management techniques and their	Adaption of the Chronic Disease Self-Management Program (CDSMP) in content and structure for implementation in psychiatric settings and for individuals with SMI	<u>Outcome measures:</u><br>- Self-reports about health functioning (general health, physical health, emotional well-being)<br>- Attitudinal measures (self-management, self-efficacy, patient-activation, internal health-locus of control, recovery assessment)<br>- Behavioral measures (healthy eating, physical activity, accessing social support, behavioral and cognitive symptom management, making better use of health care, general self-management behaviors)<br>- Emergency service use<br><u>Main findings:</u><br>- Improvement in health functioning measures, self-efficacy, patient-activation, general self-management

					application to lifestyle; Medication management and coordination of general medical and psychiatric services		behaviors and effective use of health care<br>- Attenuation of most effects in 2 months-follow-up but continued improvements in general self-management behaviors<br>- More frequent and longer booster sessions after intervention could be important for maintenance of effects
18	Goodrich et al., 2012; Kilbourne et al., 2013 [70,81]	USA	RCT	<u>Aim:</u> To examine the effects of an intervention to reduce CVD risk factors and to improve physical and mental health outcomes for individuals with bipolar disorder<br><u>Context/Setting:</u> A mental health outpatient clinic and a primary care outpatient clinic<br><u>Sample:</u> 58 individuals with bipolar disorder and >=1 CVD risk factor	- Overall intervention duration: 12 months<br>- Four two hour group-based psychoeducational self- management sessions including CVD risk in context of bipolar disorder<br>- Care management: Patient follow up by a health specialist after self-management sessions for up to 12 months; Continuing review of health behavior goals and care needs; Provided contact between	Based on Chronic Care Model, adapted using Social Cognitive Theory to focus on health behavior change	<u>Outcome measures:</u><br>*primary outcomes:*<br>- Physiological CVD risk measures (blood pressure, total cholesterol)<br>- physical health related quality of life<br>*secondary outcomes:*<br>- HDL, LDL<br>- Weight, BMI, waist circumference<br>- 10-year CVD risk (based on cholesterol, blood pressure and self-reported risk factors)<br>- Mental health related quality of life<br>- Psychiatric symptoms<br><u>Main findings:</u>

					mental health and general medical providers if needed<br>- Continuing medical education for mental health and general medical providers		- Reduced blood pressure<br>- Reduced manic symptoms<br>- No reductions of other secondary outcomes
19	Hansen et al., 2016 [71]	Denmark	One-group pre-post design	Aim: To evaluate the effects of an already developed health interventions for outpatients with schizophrenia on cardiovascular health and to examine which baseline characteristics are associated with special outcomes in the patient population.<br>Context/Setting: Two outpatient facilities treating individuals with schizophrenia.<br>Sample: 108 individuals with<br>1. newly diagnosed schizophrenia (antipsychotic drug treatment for less than 3 months)<br>2. long term ill patients (diagnosed at least 2 years prior to study)<br>in the age group 18 - 45 years, who took part in an 2-year treatment program	- Intervention duration: 15 months<br>- Intervention frequency: variable/weekly or less frequently<br>- Natural setting with wide range of patient's use of offered interventions<br>- Individual consultations with staff members, who are using motivational interviewing techniques to mobilize patient's health behavior<br>- 8 group sessions with educational topics to improve health<br>- Offers for physical exercise	Intervention is based on evidence-based practices using active awareness methods, group sessions and staff role modeling	Outcome measures:<br>- Weight, height, waist circumference, blood pressure, serum assays of blood lipids, and HbA1c measures.<br>- Information on smoking, alcohol and substance abuse<br>Main findings:<br>- No significant improvement of cardiovascular risk factors<br>- Small increases in BMI and waist circumferences<br>- High baseline BMI and being long-term ill predicted more successful outcomes<br>- Effects of newly initiated antipsychotic drug treatment in many participants could have undermined the interventional effects<br>- No correlation between number of interventions and mean reduction in outcomes

					- Awareness and encouragement of staff members to increase own physical health		- Only small percentage of patients contacted their general practitioner after being encouraged to do so
20	Happell et al., 2018 [72]	Australia	Study protocol for a RCT	<u>Aim:</u> To describe a trial implementing a nurse role to coordinate physical health care in a community mental health setting<br><u>Context/Setting:</u> Community-based mental health service<br><u>Intended Sample:</u> 80 individuals with DSM-5 diagnosis of psychosis	- Intervention frequency: Quarterly consultations and monthly telephone follow up<br>- 24 months of additional nurse-led support in the mental health service<br>- Risk management: assessment of clinical and behavioral health risk factors and consumers' self-identified goals<br>- Care coordination: supported referral to programs and services and support for treatment adherence	Intervention is grounded on the "Integrated Theory of Health Behavior Change"	<u>Intended outcome measures:</u><br>- Cardiometabolic health: clinical (BMI, total cholesterol, fasting glucose, and blood pressure) and health behavioral measures: (smoking, eating vegetables, exercise, binge drinking)<br>- Acceptability: self-reports of access, acceptability, shared decision making, quality of life<br>- Cost-effectiveness: Assessing Cost Effectiveness Prevention methodology
21	Happell, Curtis et al., 2014; Happell, Stanton et al., 2014 [73,74]	Australia	Study protocol for a RCT/ Feasibility study	<u>Aim:</u> To report the utilization of primary care and allied health referrals using an nurse-led intervention for individuals with SMI	- Referral to a specialized mental health nurse with expertise in physical health care	Nurse-led intervention based on existing framework (Integrated Theory	<u>Outcome measures:</u><br>- Medical records of physical health and primary care settings

				<u>Context/Setting:</u> Regional community mental health service<br><u>Sample:</u> 21 mental health consumers (diagnosis not mentionned)	coordination and health promotion within the setting<br>- Coordination of physical health care by identifying need for, and providing referrals to primary care as well as allied health and community-based services.<br>- Two 30-minutes consultations with health nurses at baseline and on completion; 26 weeks of intervention with additional support (frequency and duration not specified)	of Health Behavior Change); Rationale for intervention based on evidence on reduced service use and insufficient health behavior of individuals with SMI	- Individual physical health measures according to examined baseline measures<br><u>Main findings:</u><br>- Sample had high prevalence of chronic comorbid illness<br>- CHNs were helpful in health care referals, two third of the sample received referrals
22	Illinois Institute of Technology, 2022 [75]	USA	RCT	<u>Aim:</u> To investigate the effects of a peer health navigation intervention to increase physical health and wellbeing of individuals with SMI<br><u>Context/Setting:</u> Time and places<br>convenient to the person as needed	- 12 months of intervention (4 months of maintenance phase)<br>- Weekly intervention frequency<br>TAU:<br>- participants receive integrated physical	Implementation framework:<br>Intervention was developed in a community-based participatory research program; Background of	<u>Intended outcome measures:</u><br>*Outcomes:*<br>- Service engagement (participation data)<br>- Physical symptoms<br>- Blood pressure<br>- Recovery<br>- Quality of life<br>*Mediators:*

				Sample: 150 individuals with SMI (not specified)	and mental health care from their usual provider<br>Experimental:<br>- Face to face meetings between participants and peer navigators in time and places convenient to the person as needed<br>- Scheduling and attending healthcare appointments<br>- Partnering with participants on tasks that arise from appointments<br>- Health-related goal setting<br>- Taking action-steps toward health-related goals<br>-	Intervention not specified	- Personal empowerment<br>- Self-determination<br>- Perceived relationship for recovery<br>*Process measures:*<br>- Fidelity (self-report)<br>- Feasibility (self-report)<br>- Acceptability (self-report)
23	Jakobsen et al., 2017; Speyer et al., 2016; Speyer et al., 2015 [76,99,100]	Denmark	Study protocol for RCT/ RCT/ 2-year follow-up study	Aim: To investigate the effect of an intensive lifestyle intervention for individuals with schizophrenia<br>Context/Setting: Home visits and contact via phone, messages and e-mail	- Lifestyle coaching: manualized coaching to address physical inactivity, unhealthy dietary habits and smoking, and facilitating contact to	Intervention based on different theoretical models: Theory of stages of change; Motivational interviewing; Assertive	Outcome measures:<br>- 10 year risk of cardiovascular disease including modifiable and non-modifiable risk-factors (self-report)<br>- Cardiorespiratory fitness, blood parametres, waist

				Sample: 138 individuals with schizophrenia spectrum disorder + overweight.	treat somatic comorbidities<br>- Affiliation with coach (occupational therapists, physiotherapists or dieticians) for one year; In general one personal meeting per week of one hour<br>- Care coordination: one year contact with a trained psychiatric nurse; Facilitation to primary care; Frequency of contact was adjusted according to individual health needs	community treatment. Content based on Danish Health Authority guidelines	circumference, and self-reported physical activity<br>- Smoking behavior, dietary behavior, sedentary behavior, PANAS, quality of life, cognition, psychosocial functioning, and perceived stress (all self-reported)<br>- Assessment after 12 months and after 24 months<br>Main findings:<br>- Neither CHANGE intervention, nor care coordination were superior to standard treatment<br>- These findings were reported for 12-months and 24-months follow up<br>- Lack of beneficial results of the lifestyle intervention and moderate compliance with weekly meetings questions this approach for patients with schizophrenia<br>- No-difference in three conditions could be due to possible effect of the already well established danish health-care system

24	Kelly et al., 2017; Kelly et al., 2014 [77,78]	USA	Pilot Study/ RCT	Aim: To investigate the effect of an intervention aimed to improve self-management of health and healthcare for individuals with SMI<br>Context/Setting: An Outpatient community mental health agency for adults with SMI<br>Sample: 76 individuals with SMI (schizophrenia, schizo-affective disorder, bipolar disorder, or major depression)	- 6 months manualized intervention delivered by peer-health navigators; About one in-person and one phone contact per month<br>- Support to increase access to and use of health care, improve healthcare experience, and foster self-management<br>- Optional ongoing support and boosters as needed	Based on Model for Vulnerable Populations	Outcome measures:<br>- Service engagement and working alliance (self-report)<br>- Intervention fidelity and intensity (self-report)<br>- Self-reported health service utilization<br>- Satisfaction with primary care provider<br>- Self-management attitudes and behaviors (self-report)<br>- Self-reported routine health screening<br>- Medical diagnosis (self-reported)<br>- Pain<br>Main findings:<br>- Increased detection of undiagnosed chronic diseases<br>- Increased self-management confidence of participants (attitudinal and behavioral)<br>- Improvements in the location, frequency, and quality of relationships with routine care<br>- Decrease in emergency service use
25	Kilbourne, Goodrich et	USA	Pilot RCT	Aim: To examine a bipolar disorder medical care model	- Four two hour group-based	Based on the Bipolar Disorder	Outcome measures (only self-reports):

	al., 2008; Kilbourne, Post et al., 2008 [83,84]			to improve medical and psychiatric outcomes<br>Context/Setting: Department of Veterans Affairs mental health facility<br>Sample: 27 individuals with bipolar disorder and >= 1 CVD risk factor	psychoeducational self- management sessions including CVD risk in context of bipolar disorder<br>- Care-management: nurse care manager provided contact between providers and patients for up to six months; Monthly phone-calls to review patient's progress<br>- Series of one-hour continuing medical education for mental health and general medical providers	Collaborative Chronic Care Model, an adaption of the Chronic Care Model with focus on CVD risk reduction	- Physical and mental health-related quality of life<br>- Global functioning<br>- Bipolar disorder symptoms<br>- Sociodemographic characteristics<br>- Access to care<br>Main findings:<br>- Improved physical health-related quality of life<br>- Small, non-significant increases in other outcome measures<br>- Intervention has the potential to be adopted in settings with few resources
26	Kilbourne et al., 2017; Kilbourne et al., 2014 [79,80]	USA	RCT	Aim: To examine the effects of the Life Goals-Collaborative Care Model on physical health in individuals with SMI within 12 months<br>Context/Setting: Veterans Affairs urban outpatient mental health clinic<br>Sample: 146 individuals with SMI (schizophrenia, bipolar disorder, or major depressive disorder) and >=1	- Five 90-minutes group-based psychoeducational self- management sessions focused on reducing psychiatric symptoms by promoting healthy behaviors.<br>- Care-management: : Patient follow up by a health specialist for 6 months; Six monthly	Life Goals Collaborative Care: Based on collaborative Care Model with adaption to CVD risk reduction	Outcome measures:<br>*primary outcomes:*<br>- Health related quality of life<br>*secondary outcomes:*<br>- CVD risk measures (clinical and self-reports)<br>- Psychiatric symptoms<br>- 10-year CVD risk (clinical measures and self-reports)<br>- Waist circumference<br>Main findings:

				cardiovascular disease risk factor	contacts of 20 minutes. Continuing review of health behavior goals and care needs, provided contact between mental health and primary care providers if needed<br>- Dissemination of patient's care plan to primary care and mental health providers after last care management contact. Continuing medical education for mental health and general medical providers		- Greater improvement in physical health-related quality of life after 12 months<br>- No improvements in secondary outcomes<br>- Outcomes remain suboptimal for persons with chronic mental disorders
27	Kilbourne et al., 2015 [82]	USA	RCT	Aim: To examine the effectiveness of a standard versus enhanced implementation strategy of Life Goals-Collaborative Care Model (LG-CC) for bipolar disorder<br>Context/Setting: Community based mental health and primary care clinics	- Life Goals Collaborative Care Intervention cf. study no. 26<br>- Standard REP: Intervention training and technical assistance<br>- Enhanced REP: Customization of intervention based on	*Implementation framework:* REP: Based on Rogers' Diffusion of Innovations and Social Learning Theories / Enhancement to promote use of Collaborative Care	Outcome measures:<br>- Mood symptoms<br>- Health related quality of life<br>- Functioning (self-report)<br>Main findings:<br>- Enhanced REP was not associated with improved mental or physical health-related quality of life, reduced functional impairment, manic symptom

				<u>Sample:</u> 177 individuals with bipolar disorder	input from local site providers to fit local needs using interviews and focus groups; ongoing facilitation for 12 months	Models for mental health conditions<br>*Intervention framework:* Life Goals Collaborative Care: Based on collaborative Care Model with adaption to CVD risk reduction	severity, or greater likelihood of depression remission.
28	Kim et al., 2017 [85]	USA	Mixed-methods study	<u>Aim:</u> To examine the implementation and outcomes of an integration pilot program for individuals with SMI and co-occurring chronic conditions<br><u>Context/Setting:</u> Partnership project between behavioral health and physical health managed care organizations<br><u>Sample:</u> 857 to 4788 individuals with SMI (schizophrenia, major mood disorder, or borderline personality disorder)	- Overall intervention duration: 2 years<br>- Employment of navigators (different professional backgrounds) with following tasks:<br>- Comprehensive care management: at least monthly sessions with participants to review medication adherence and use of health care services<br>- Care coordination: facilitating access to and monitoring of services to manage chronic conditions	HealthChoices HealthConnections Program: No explicit model described in the study; Based on empirical eclectic findings	<u>Outcome measures:</u><br>*administrative data:*<br>- health care claims (ED visits, hospitalizations, and readmissions)<br>- enrollment data<br>*interviews and focus groups with providers and consumers:*<br>- factors and strategies that facilitated or posed challenges to implementation<br><u>Main findings:</u><br>- Improvements in ED visits limited to one intervention region<br>- No differences in hospitalizations and readmissions<br>- Value of nurse navigators confirmed by providers

					- Health promotion to address health and wellness goals<br>- Transitional care/ follow-up<br>- Individual and family support<br>- Referral to community and social support services		- Current state of health information technology is not yet sufficiently developed
29	Lewis et al., 2020 [87]	Australia	Study protocol for a RCT	<u>Aim:</u> To test a co-produced, nurse-led intervention delivered with general practitioners to reduce absolute CVD risk<br><u>Context/Setting:</u> Primary care setting including general practices and community health centers<br><u>Intended Sample:</u> 252 individuals with SMI (schizophrenia, bipolar disorder, major depression, schizophrenia or other SMI identified) aged 35 - 74	- Delivery of intervention for 12 months<br>- Baseline assessment of absolute cardiovascular disease risk; Discussion of results and creation of health plan with research nurse in conjunction with participants' GP<br>- Support to improve lifestyle changes<br>- Assertive support: weekly phone contact with study nurse to review participants' health goals; up to 5	Integrates guideline level cardiovascular disease preventive care with key principles of Assertive Community Treatment and Motivational Interviewing. Iterative co-production with people with lived-experience of SMI in 6 steps including piloting.	<u>Intended outcome measures:</u><br>*primary outcomes:*<br>- 5-year absolute cardiovascular disease risk (score calculation)<br>*secondary outcomes:*<br>- 6-month absolute cardiovascular disease risk (score calculation)<br>- Blood pressure, lipids, triglycerides, HbA1c<br>- BMI<br>- Quality of life<br>- Physical activity (self-report)<br>- Motivation to change health behavior<br>- Medication adherence (self-report)<br>- Alcohol use (self-report)

					in-person appointments		- Hospitalization at 6 and 12 months
30	Meepring et al., 2018 [89]	Thailand	Quasi-experimental pre-post study	<u>Aim:</u> To examine the effects of an intervention to improve physical health and reported health behaviors<br><u>Context/Setting:</u> Outpatient clinic attached to a regional psychiatric hospital<br><u>Sample:</u> 105 individuals with SMI (including schizophrenia, schizoaffective disorder, or bipolar disorder) aged 18 – 65	- 12 months of intervention<br>- Assessment of patients' physical state and health behaviors<br>- 4 to 6 appointments of 20 min with mental health nurses over the 1-year duration<br>- Development and evaluation of individualized physical health-care plans using motivational interview techniques (especially brokerage of existing health care services and information about lifestyle behaviors for health promotion)	Cultural adaptation of the "Health Improvement Profile" (HIP), an evidence-based physical health risk assessment with subsequent intervention	<u>Outcome measures:</u><br>- Items of the HIP-T assessment tool (based on self-reports) (health/lifestyle behaviours and potential health risks, e.g., sleep behaviour, smoking status, alcohol intake, exercise behaviour and others)<br>- Blood pressure, pulse, weight, height and waist circumference (measured)<br><u>Main findings:</u><br>- Significant improvements in mean BMI and bodyweight<br>- Significant reduction in red-flagged HIP-T items (e.g., sleep =< 3 hours)<br>- Baseline physical health and health behaviors were better than in some other Asian and Western countries
31	Nover, 2013 [90]	USA	Qualitative study	<u>Aim:</u> To explore participants experiences with an augmented care program<br><u>Context/Setting:</u> Primary care clinic with medication-only psychiatric service	- Delivery of intervention for 12 months<br>- Employment of an additional social worker and an	Augmented services / CalMEND CPCI program (no explicit model or theory mentioned)	<u>Outcome measures:</u><br>- Open-ended questions based on the review of the literature and elements of the intervention<br><u>Main findings:</u>

				Sample: 9 individuals with SMI (recurrent major depressive disorder, bipolar disorder, or schizophrenic disorder) with diagnosis of, or risk factors for, hypertension, coronary artery disease, dyslipidemia, or diabetes	additional mental health nurse who provides care coordination, supportive and educational groups (self-esteem, exercise, weight loss, and diabetes) and individual weight loss support<br>- Identification and consistent screening of risk factors of physical illness		- Dissatisfaction with usual care prior to intervention is often perceived as a passive process<br>- Some participants reported non-adherence in usual care because of feeling, that their opinions were not valued<br>- Increasing patient-satisfaction through the self-management focus and supportive environment of the intervention<br>-
32	Ohlsen et al., 2005 [91]	England	Quasi-experimental pre-post study	Aim: To describe set up and implementation of a service to monitor and improve physical health in people with SMI<br>Context/Setting: Nurse advisor in an unfamiliar community mental health team<br>Sample: 79 individuals with SMI (schizophrenia, bipolar disorder, schizo-affective disorder)	- Overall intervention duration unclear<br>- No distinct information on intervention frequency (groups were held on a weekly basis)<br>- Employment of a nurse advisor to identify physical health needs and to promote access to health care<br>- Creation of working agreements/ care	Summary of evidence about reasons for poor physical health in SMI; no explicit theoretical framework for intervention mentioned	Outcome measures:<br>- Cardiovascular health parametres<br>- Current medication and medication history, assessment of medication side-effects (self-report)<br>- Demographic and biographic details<br>- Self-reported behavioral health parameters (weight gain, medication, substance use)<br>- self-esteem assessment<br>Main findings:

					networks with health care providers through nurse advisor<br>- Initial baseline assessment and screening of different physical and behavioral health parameters<br>- Referral to weight management and exercise groups<br>- Referral to general practitioners and appropriate specialists<br>- Weight management and dietary support		- Improvement in quality of diet<br>- Improvement in self-esteem<br>- Majority of patients lost weight and attended weight management or physical activity group they did not attend before
33	Pastore et al., 2013 [92]	USA	Pilot One-group pre-post study	Aim: To evaluate utilization and functional status before and after implementation of an enhanced care model<br>Context/Setting: Primary care facility in a hospital-based health system<br>Sample: 16 individuals with SMI (Psychotic disorders, mood disorders, bipolar disorder)	- Assessment period of 9 months<br>- Intervention frequency not specified<br>- Consistent behavioral health liaison through a registered nurse which assisted in access and coordination of care and health education	Based on the Assertive Community Treatment model	Outcome measures:<br>- Number of primary care visits, missed appointments, emergency department utilization, and receipt of health promotion initiatives (mammograms, cervical cytology, fasting lipid profiles, prostate specific antigen, blood glucose, and colorectal screening)<br>- Health-related quality of life

					- Educational sessions for primary care staff (Project overview; SMI; Increased mortality and morbidity; Barriers to primary care services; Stigma)<br>- Use of hand held medical record to facilitate communication and coordination of care		- Acceptability and utilization of health passport (self-report and participation data)<br><u>Main findings:</u><br>- Reductions in number of missed appointments<br>- Care continuity was identified as an important factor both by participants and staff members<br>- No significant changes in ER utilization and self reported quality of life<br>- Hand held medical record was not used by participants
34	Pratt et al., 2013 [93]	USA	Pilot One-group pre-post study	<u>Aim:</u> To examine the feasibility and effectiveness of an telehealth intervention<br><u>Context/Setting:</u> Telehealth device at homes of participants<br><u>Sample:</u> 70 individuals with SMI (PTSD, schizophrenia, schizoaffective disorder, major depressive disorder, or bipolar disorder) and at chronic medical conditions	- Participants received a electronic device with a LCD Screen<br>- Daily telehealth sessions (5-10 min) for 6 months with questions regarding symptoms, health indicators, self-management, and health behaviors supported by a nurse at a mental health center	Empirical evidence of telehealth from other vulnerable patient groups experiencing difficulties in access of health care; Based on eclectic empirical evidence	<u>Outcome measures:</u><br>*Behavioral measures:*<br>- Rate of participant adherence (participation data)<br>*Self-reported measures:*<br>- Disease self-efficacy<br>- Knowledge of mental illness<br>- Subjective physical and emotional health status<br>- Psychiatric symptoms<br>- Health service use<br>- Objective health measures: Blood pressure, blood

					- Coaching of users at beginning of study at their homes<br>- Risk-profile based an health questions and contact via phone or text message by health nurse to provide advice<br>- When necessary, nurse provided home visits or accompanied to medical appointments		glucose, weight (self-measured)<br>Main findings:<br>- High adherence and willingness to continue using the device<br>- Increased perception of disease-related knowledge<br>- Significant improvement in self-efficacy for managing depression and diastolic blood pressure
35	Rozing et al., 2021 [94]	Denmark	Study protocol for a cluster randomized controlled trial	Aim: To evaluate the feasibility of a coordinated co-produced care program in the general practice setting to improve quality of life and reduce mortality for individuals with SMI<br>Context/Setting: 12 general practices recruiting community-dwelling patients<br>Sample: Individuals with SMI (psychotic disorder, bipolar disorder, severe depressive disorder)	- Prolonged clinical consultations, focusing on somatic health problems, lasting up to 45 min<br>- Educational course for GPs and practice staff<br>- Regionalized handbook for GPs to coordinate trans-sectional care for patients<br>- Establishment of a follow-up plan with patients<br>- 6-month study period	SOFIA-Intervention combines evidence-based clinical, and social knowledge; Perspectives of all involved parties will be integrated in a participatory co-design process	Intended outcome measures:<br>- Implementability of the intervention<br>- Patient retention<br>- Quality of life<br>- Practices electronic medical records (CPR-number, sex, age, ICPC-2 codes and ICD-10 codes)<br>- Mortality and morbidity data<br>- Mobility, self-care activities, pain/discomofort, anxiety and depression<br>- Medication<br>- Biochemical and biometric data

36	Smith et al, 2007 [97,98]	UK	One-group pre-post study	Aim: To examine if a nurse-led physical health promotion programme could modify lifestyle factors in individuals with SMI<br>Context/Setting: Seven mental health trusts with different socio-economic and geographical characteristics.<br>Sample: 966 individuals with SMI (schizophrenia, schizoaffective disorder, severe affective disorders)	- Intervention held by a registered mental nurse<br>- Minimum of six consultations over a maximum of 2 years<br>- Baseline check-up of health and health behavior characteristics and discussion about results<br>- Provision of a weight management and a physical activity group<br>- Referral to GP for further physical health care<br>- Referral to specialist for further physical health care<br>- Change of medication	Not specified	Outcome measures:<br>- Cardiovascular risk factors: BMI; blood pressure; smoking; alcoholm; physical activity (self-report), diet<br>- Self-esteem<br>Main findings:<br>- Cardiovascular risk factors were reduced by the nurse-led intervention (significant effects for behavioral measures and self-esteem)<br>- Persistence of effects for at least 2 years
37	van der Voort et al., 2015 [101]	Netherlands	Cluster RCT	Aim: To study the effects of Collaborative Care on functional impairment and quality of life, and to examine to which extend the severity of depressive symptoms mediates functional recovery and quality of life	- Intervention for 12 months<br>- Creation of collaborative care teams, including the patient, a relative of the patient, a general	Based on eclectic empirical evidence of collaborative care in patients with bipolar disorder	Outcome measures:<br>- Functioning (self-report)<br>- Quality of life (self-report)<br>Main findings:<br>- Significant improvement in functioning (overall

				Context/Setting: 16 mental health outpatient clinics<br>Sample: 56 individuals with bipolar disorder	nurse and the psychiatrist.<br>- At least 3 team meetings per year<br>- Coordination of care provided by the nurse<br>- Systematical assessment of care needs and subsequential treatment plan<br>- Relapse prevention plan<br>- Psycho-education groups<br>- Problem-solving therapy provided by nurses to enhance mood		functioning, autonomy, cognition, leisure time)<br>- No differences found in improvements in overall and health related quality of life<br>- Improvements in the subdomain of physical health<br>- Reducing depression severity accounts for half of the improvement in functioning
38	Velligan et al., 2013 [102]	USA	Case control study (three group quasi experimental design)	Aim: To investigate an intervention to improve compliance with guidelines for monitoring metabolic syndrome for people<br>Context/Setting: Mental health outpatient clinic<br>Sample: Individuals with SMI (not specified)	- Chart observation period of 18 months<br>- Removing barriers to ensure diagnostics (Staff education; Facilitation of delivery system interventions and procedures; Record of all data in one place)<br>- Creation of a new position: Medical	Empirical evidence of importance of guideline application in metabolic syndrome<br>*Implementation framework:* Implementation of Change Model	Outcome measures:<br>- Indices for guideline compliance (e.g., needed labs ordered, documentation of clinical parametres)<br>Main findings:<br>- significant compliance improvement in:<br>- needed labs ordered<br>- documentation of blood pressure, BMI and waist circumference

					assistant for metabolic monitoring (responsible for collection of participation data)		- no change in physician action on out of range values

Note: n = 38 studies represented in 51 articles with focus to promote access to physical health care.

[1] If intervention is not a one-group design, sample sizes describe the number of indivuals included in the intervention group.
