# Supplementary material for: Interventions to Promote the Utilization of Physical Health Care for People with Severe Mental Illness: A Scoping Review
Source: Int J Environ Res Public Health. 2022 Dec 22;20(1):126. doi: 10.3390/ijerph20010126 (PMC9819522; doi:10.3390/ijerph20010126)
Supplement: Supplementary file 1 [file ijerph-20-00126-s001.zip › Table 1-Table 4_V3.pdf]

Table 1. Types and content of the interventions to promote the utilization of physical health services for people with a SMI. (Note: Additional interventions not predominantly targeting the utilization of physical health services are written in italics; listed types and content of interventions are not mutually exclusive).

**Type/Content of the Study Intervention**	**Studies (Study Identification Number from Table S7, See Supplementary Materials)**	**Number of Studies (n = 38)**
Delivery involves individual setting	1, 2, 3, 5, 6, 7, 8, 9, 10, 11, 12, 13, 14, 15, 16, 17, 18, 19, 20, 21, 22, 23, 24, 25, 26, 27, 28, 29, 30, 31, 32, 33, 34, 35, 36, 37	37
Delivery involves group setting	1, 4, 5, 6, 7, 13, 15, 17, 18, 19, 25, 26, 27, 31, 32, 36	17
Phone maintenance	1, 29	2
App-based intervention	34	1
Peer-led intervention	15, 17, 22, 24	4
Peer-specialist involvement	4, 13	2
Care-management	2, 3, 5, 6, 8, 9, 11, 12, 13, 14, 16, 18, 20, 21, 22, 23, 24, 25, 26, 27, 28, 31, 32, 33, 35, 36, 37	27
Self-management training	1, 4, 6, 7, 10, 11, 12, 13, 14, 15, 16, 17, 18, 20, 22, 24, 25, 26, 27, 28, 29, 30, 34	23
Physical health education	4, 5, 11, 13, 14, 15, 17, 18, 19, 23, 25, 26, 27, 28, 31, 33	16
Health screening and monitoring	5, 6, 7, 10, 11, 12, 20, 21, 29, 30, 31, 32, 34, 35, 36, 37	16
Distinct training to improve health care utilization	1, 2, 4, 5, 7, 8, 13, 15, 16, 17, 23, 24, 30	13
Care-plan development	7, 22, 25, 26, 27, 29, 30, 35, 37	9
Motivational support	2, 7, 12, 14, 16, 19, 30	7
Treatment adherence support	2, 7, 12, 15, 17, 20	6
Problem solving training	1, 7, 14, 22, 37	5
Improvement of health care information interface	3, 11, 33, 38	4
Empowerment	3	1
Stigma-reduction	27	1
Structured doctor and nurse visits	3	1
Lifestyle changes	1, 12, 15, 17, 19, 23, 29, 30, 31, 32, 34, 36	12
Mental health education	1, 5, 6, 9, 17, 18, 25, 26, 27, 37	10
Staff training	3, 4, 18, 25, 26, 27, 33, 35, 38	9
Wellness enhancement	5, 13, 28, 31	4
Involvement of social network	8, 28, 37	3
Crisis intervention	8	1
Critical appraisal of medication	36	1
Local implementation customization of intervention	38	1
Support in daily living	3	1

Table 2. Reported outcome measures in the studies including interventions to promote the utilization of physical health care for people with a SMI.

Outcome Measure	Studies (Study Identification Number from Table S7, See Supplementary Materials)	Number of Studies (n = 38)
Self-report based data	1, 2, 3, 4, 5, 6, 7, 9, 10, 11, 12, 13, 14, 15, 16, 17, 18, 19, 20, 22, 23, 24, 25, 26, 27, 28, 29, 30, 31, 32, 33, 34, 35, 36, 37	35
Thereof: no other data-sources than self-report based data	10, 13, 15, 17, 24, 25, 27, 31, 37	9
Physiological measures	1, 7, 9, 12, 14, 18, 19, 20, 21, 22, 23, 26, 29, 30, 32, 34, 35, 36	18
Administrative data	3, 8, 11, 16, 21, 28, 29, 33, 35, 38	10
Participation data (e.g., attendance rate)	6, 11, 22, 33, 34	5
Behavioral assessment	2, 4	2

Table 3. Study authors' interpretation of the success of the interventions to promote the utilization of physical health care for people with a SMI.

**Study Authors' Interpretation of the Intervention Success**	**Studies (Study Identification Number from Table S7, See Supplementary Materials)**	**Number of Studies (n = 33)**
Improvement of other physical health-behavior related outcomes, besides service utilization	1, 2, 4, 6, 11, 13, 14, 15, 17, 24, 30, 32, 34, 37	14
Improvement in physical health outcomes	2, 5, 9, 12, 14, 15, 18, 25, 26, 30, 32, 36, 37	13
Improvement in health service utilization	3, 5, 6, 9, 11, 17, 21, 24, 33, 38	10
Improvement in mental health outcomes	1, 2, 3, 5, 15, 16, 18, 32, 36, 37	10
No positive physical health related outcomes at all	10, 19, 23, 27, 28, 31	6
Reduction of emergency department use	5, 6, 8, 24	4

Table 4. Theoretical rationale or model of interventions to promote the utilization of physical health care for people with a SMI.

Theoretical Rationale or Model	Studies (Study Identification Number from Table S7, See Supplementary Materials)	Number of Studies (n = 38)
Based on evidence-based intervention (s)	1, 2, 4, 5, 6, 10, 11, 12, 13, 14, 15, 16, 17, 19, 30, 35, 37	17
Adaption of a care model or framework of healthcare	3, 5, 7, 9, 12, 18, 23, 24, 25, 26, 27, 29, 33	13
Based on established theory	1, 2, 14, 18, 20, 21, 23	7
Use of a study implementation framework	10, 22, 27, 29, 35, 38	6
No distinct model or theoretical rationale	8, 22, 28, 31, 36	5
Based on eclectic empirical evidence	21, 32, 34, 38	4
