# Supplementary material for: Interventions to Promote the Utilization of Physical Health Care for People with Severe Mental Illness: A Scoping Review
Source: Int J Environ Res Public Health. 2022 Dec 22;20(1):126. doi: 10.3390/ijerph20010126 (PMC9819522; doi:10.3390/ijerph20010126)
Supplement: Supplementary file 1 [file ijerph-20-00126-s001.zip › Table S1-Table S6.pdf]

Supplementary Table S1: Search strategy for Web of Science Core Collection

Step number	Search term entered into Web of Science	Results
1	AB=(“promotion”) OR TI=(“promotion”)	148364
2	AB=(“facilitation”) OR TI=(“facilitation”)	39140
3	AB=(“health nurse”) OR TI=(“health nurse”)	1534
4	AB=(“nurse-led”) OR TI=(“nurse-led”)	4534
5	AB=(“self-management”) OR TI=(“self-management”)	24769
6	AB=(“empowerment”) OR TI=(“empowerment”)	30159
7	AB=(“health literacy”) OR TI=(“health literacy”)	11706
8	AB=(“chronic disease management”) OR TI=(“chronic disease management”)	2191
9	AB=(“general practitioner management”) OR TI=(“general practitioner management”)	40
10	AB=(“barrier support”) OR TI=(“barrier support”)	9
11	AB=(“care manager”) OR TI=(“care manager”)	437
12	AB=(“care management”) OR TI=(“care management”)	7775
13	AB=(“case manager”) OR TI=(“case manager”)	1099
14	AB=(“case management”) OR TI=(“case management”)	9804
15	AB=(“patient activation”) OR TI=(“patient activation”)	1260
16	AB=(“self efficacy”) OR TI=(“self efficacy”)	51635
17	AB=(“coordination of care”) OR TI=(“coordination of care”)	1600
18	AB=(“care coordination”) OR TI=(“care coordination”)	4160
19	AB=(“coordinated care”) OR TI=(“coordinated care”)	1217
20	AB=(“integration of care”) OR TI=(“integration of care”)	353
21	AB=(“integrated care”) OR TI=(“integrated care”)	5962
22	AB=(“care integration”) OR TI=(“care integration”)	633
23	#1 OR #2 OR #3 OR #4 OR #5 OR #6 OR #7 OR #8 OR #9 OR #10 OR #11 OR #12 OR #13 OR #14 OR #15 OR #16 OR #17 OR #18 OR #19 OR #20 OR #21 OR #22	332695
24	AB=(“primary” AND "care") OR TI=(“primary” AND "care")	239608
25	AB=(“physical” AND "care") OR TI=(“physical” AND "care")	80082
26	AB=(“somatic” AND "care") OR TI=(“somatic” AND "care")	4665
27	#24 OR #25 OR #26	305283
28	AB=(“bipolar") OR TI=(“bipolar”)	109679
29	AB=(“borderline") OR TI=(“borderline”)	46435
30	AB=(“major depression") OR TI=(“major depression”)	25573
31	AB=(“psychosis") OR TI=(“psychosis”)	47924
32	AB=(“psychotic") OR TI=(“psychotic”)	36184
33	AB=(“schizophrenia spectrum disorder") OR TI=(“schizophrenia spectrum disorder”)	896
34	AB=(“schizoaffective") OR TI=(“schizoaffective”)	5830
35	AB=(“schizo-affective") OR TI=(“schizo-affective”)	297
36	AB=(“schizophrenia") OR TI=(“schizophrenia”)	141614
37	AB=(“schizophrenic") OR TI=(“schizophrenic”)	25842

38	AB=(“persistent mental illness") OR TI=(“persistent mental illness”)	532
39	AB=(“serious mental disorder") OR TI=(“serious mental disorder”)	171
40	AB=(“serious mental illness") OR TI=(“serious mental illness”)	4060
41	AB=(“serious psychiatric disorder") OR TI=(“serious psychiatric disorder”)	92
42	AB=(“serious psychiatric illness") OR TI=(“serious psychiatric illness”)	52
43	AB=(“severe mental disorder") OR TI=(“severe mental disorder”)	501
44	AB=(“severe mental illness") OR TI=(“severe mental illness”)	5268
45	AB=(“severe psychiatric disorder") OR TI=(“severe psychiatric disorder”)	258
46	AB=(“severe psychiatric illness") OR TI=(“severe psychiatric illness”)	156
47	#28 OR #29 OR #30 OR #31 OR #32 OR #33 OR #34 OR #35 OR #36 OR #37 OR #38 OR #39 OR #40 OR #41 OR #42 OR #43 OR #44 OR #45 OR #46	376970
48	#23 AND #27 AND #47	654
49	2022 or 2021 or 2020 or 2019 or 2018 or 2017 or 2016 or 2015 or 2014 or 2013 or 2012 or 2011 or 2010 or 2009 or 2008 or 2007 or 2006 or 2005 or 2004 or 2003 or 2002 or 2001 or 2000 (Publication Years) and English or German (Languages)	626

Note: Run date = 20/04/2022

Supplementary Table S2: Search strategy for PubMED

Step number	Search term entered into PubMED	Results
1	"Promotion"[Title/Abstract]	111732
2	"facilitation"[Title/Abstract]	32430
3	"health nurse"[Title/Abstract]	3210
4	"nurse-led"[Title/Abstract]	4514
5	"self-management"[Title/Abstract]	23715
6	"empowerment"[Title/Abstract]	15276
7	"health literacy"[Title/Abstract]	11888
8	"chronic disease management"[Title/Abstract]	2876
9	"general practitioner management"[Title/Abstract]	39
10	"barrier support"[Title/Abstract]	1
11	"care manager"[Title/Abstract]	610
12	"care management"[Title/Abstract]	9981
13	"case manager"[Title/Abstract]	1666
14	"case management"[Title/Abstract]	11989
15	"patient activation"[Title/Abstract]	1272
16	"self efficacy"[Title/Abstract]	34507
17	"coordination of care"[Title/Abstract]	1970
18	"care coordination"[Title/Abstract]	4916
19	"coordinated care"[Title/Abstract]	1520
20	"integration of care"[Title/Abstract]	390
21	"integrated care"[Title/Abstract]	6174
22	"care integration"[Title/Abstract]	687
23	#1 OR #2 OR #3 OR #4 OR #5 OR #6 OR #7 OR #8 OR #9 OR #10 OR #11 OR #12 OR #13 OR #14 OR #15 OR #16 OR #17 OR #18 OR #19 OR #20 OR #21 OR #22	262568
24	("primary"[Title/Abstract] AND "care"[Title/Abstract])	283020
25	("physical"[Title/Abstract] AND "care"[Title/Abstract])	95887
26	("somatic"[Title/Abstract] AND "care"[Title/Abstract])	5649
27	#24 OR #25 OR #26	360787
28	"bipolar"[Title/Abstract]	69849
29	"borderline"[Title/Abstract]	46566
30	"major depression"[Title/Abstract]	25585
31	"psychosis"[Title/Abstract]	43108
32	"psychotic"[Title/Abstract]	37156
33	"schizophrenia spectrum disorder"[Title/Abstract]	905
34	"schizoaffective"[Title/Abstract]	6367
35	"schizo-affective"[Title/Abstract]	354
36	"schizophrenia"[Title/Abstract]	123432
37	"schizophrenic"[Title/Abstract]	27449

38	"persistent mental illness"[Title/Abstract]	519
39	"serious mental disorder"[Title/Abstract]	156
40	"serious mental illness"[Title/Abstract]	3966
41	"serious psychiatric disorder"[Title/Abstract]	110
42	"serious psychiatric illness"[Title/Abstract]	73
43	"severe mental disorder"[Title/Abstract]	557
44	"severe mental illness"[Title/Abstract]	5048
45	"severe psychiatric disorder"[Title/Abstract]	278
46	"severe psychiatric illness"[Title/Abstract]	165
47	#28 OR #29 OR #30 OR #31 OR #32 OR #33 OR #34 OR #35 OR #36 OR #37 OR #38 OR #39 OR #40 OR #41 OR #42 OR #43 OR #44 OR #45 OR #46	303771
48	#23 AND #27 AND #47	796
49	(english[Filter] OR german[Filter]) AND (2000:2022[pdat])	765

Note: Run date = 20/04/2022

Supplementary Table S3: Search strategy for easy.dans.knaw.nl

Step number	Search strategy for https://easy.dans.knaw.nl	Results
1	(Promotion OR facilitation OR health nurse OR nurse-led OR self-management OR empowerment OR health literacy OR chronic disease management OR general practitioner management OR care manager OR care management OR case manager OR case management OR patient activation OR self efficacy OR coordination of care OR care coordination OR coordinated care OR integration of care OR integrated care OR care integration) AND (bipolar OR borderline OR major depression OR psychosis OR psychotic OR schizophrenia spectrum disorder OR schizoaffective OR schizo-affective OR schizophrenia OR schizophrenic OR persistent mental illness OR serious mental disorder OR serious mental illness OR serious psychiatric disorder OR serious psychiatric illness OR severe mental disorder OR severe mental illness OR severe psychiatric disorder OR severe psychiatric illness) AND ((primary AND care) OR (physical AND care) OR (somatic AND care))	124

Note: Run date = 13/06/2022

Supplementary Table S4: Search strategy for clinicaltrials.gov

Step number	Search strategy for https://clinicaltrials.gov	Results
1	Other terms: "primary care" OR "physical care" OR "somatic care"<br>Condition or disease: "psychotic" OR "bipolar" OR “borderline” OR "major depression" OR "serious mental illness" OR "severe mental illness"<br>Intervention/treatment: “Promotion” OR “facilitation” OR "health nurse" OR "nurse-led" OR "empowerment" OR "self-management" OR "health literacy" OR "chronic disease management" OR "care manager" OR "care management	141
2	Other terms: "primary care" OR "physical care" OR "somatic care"<br>Condition or disease: "psychotic" OR "bipolar" OR “borderline” OR "major depression" OR "serious mental illness" OR "severe mental illness"<br>Intervention/treatment: "case manager" OR "case management" OR "patient activation" OR "self efficacy" OR "care coordination" OR "coordinated care" OR "integrated care" OR "care integration"	120
3	Removing duplicates + limit 2000-2022	188

Note: Run date = 20/06/2022

Supplementary Table S5: Search strategy for trialsearch.who.int

Step number	Search strategy for https://trialsearch.who.int/Default.aspx	Results
1	(Promotion OR facilitation OR health nurse OR nurse-led OR self-management OR empowerment OR health literacy OR chronic disease management OR general practitioner management OR care manager OR care management OR case manager OR case management OR patient activation OR self efficacy OR coordination of care OR care coordination OR coordinated care OR integration of care OR integrated care OR care integration) AND (bipolar OR borderline OR major depression OR psychosis OR psychotic OR schizophrenia spectrum disorder OR schizoaffective OR schizo-affective OR schizophrenia OR schizophrenic OR persistent mental illness OR serious mental disorder OR serious mental illness OR serious psychiatric disorder OR serious psychiatric illness OR severe mental disorder OR severe mental illness OR severe psychiatric disorder OR severe psychiatric illness) AND ((primary AND care) OR (physical AND care) OR (somatic AND care))	66

Note: Run date = 27/06/2022

Supplementary Table S6: Search strategy for catalog.crl.edu/

Step number	Search strategy for http://catalog.crl.edu/	Results
1	(promotion OR facilitation OR empowerment OR activation OR literacy OR efficacy OR manag* OR coordinat* OR integrat* OR nurse) AND (major OR bipolar OR borderline OR schizo* OR psych* OR mental) AND care	11

Note: Run date = 05/07/2022
