# Supplementary figures and images for: Interventions to Promote the Utilization of Physical Health Care for People with Severe Mental Illness: A Scoping Review
Source: Int J Environ Res Public Health. 2022 Dec 22;20(1):126. doi: 10.3390/ijerph20010126 (PMC9819522; doi:10.3390/ijerph20010126)

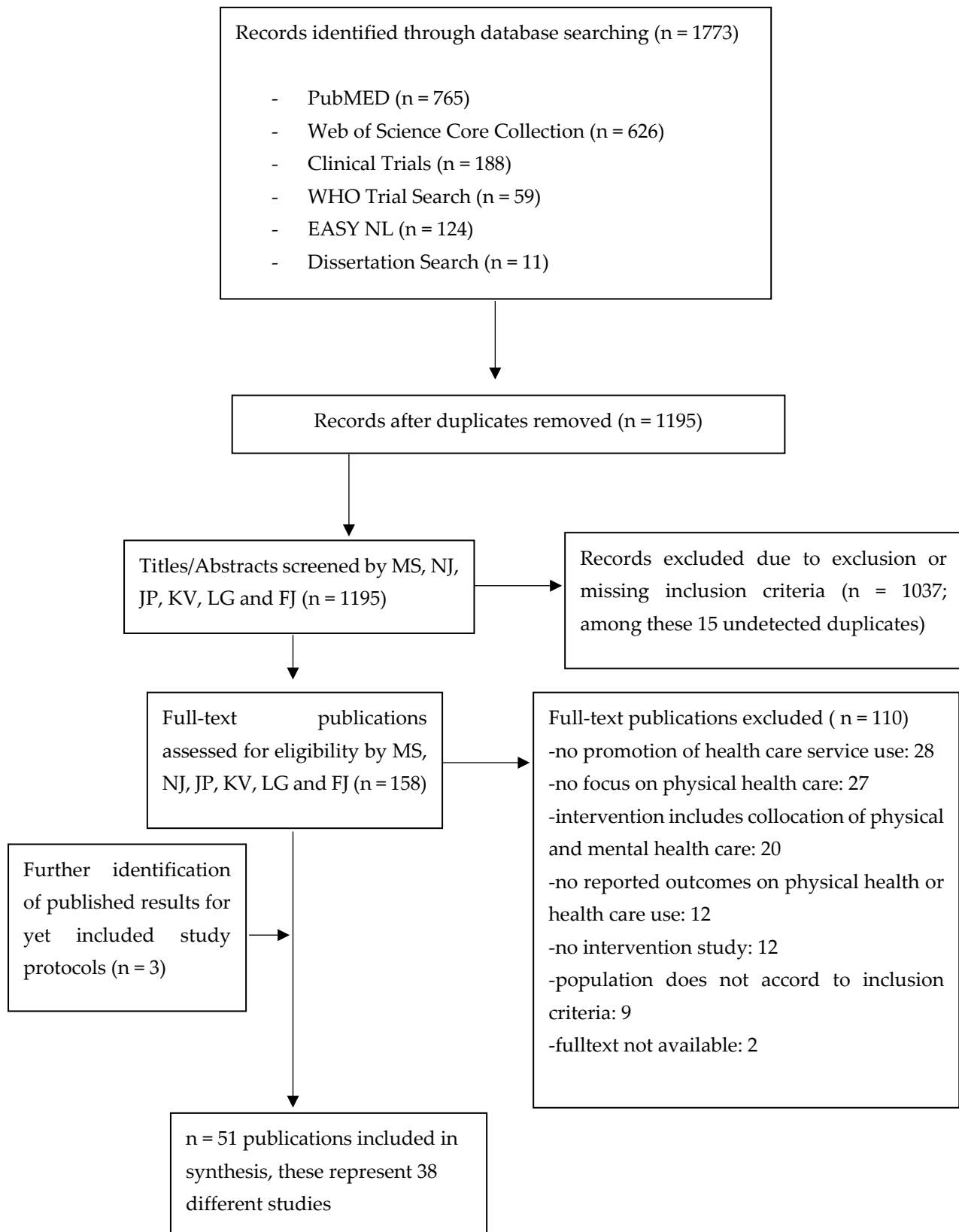

**Figure 1.** Study selection and exclusion process (PRISMA flow diagram)

Supplement: Supplementary file 1 [file ijerph-20-00126-s001.zip › Figure 1 PRISMA Flowchart.pdf]
